# Pharmacologic Treatment of Hypertension in Older Adults

**DOI:** 10.1016/j.cger.2024.04.004

**Published:** 2024-11

**Authors:** Oliver M. Todd, Matthew Knight, Joshua A. Jacobs, Catherine G. Derington, James P. Sheppard, Adam P. Bress

**Affiliations:** a Academic Unit for Ageing and Stroke Research, University of Leeds, Leeds, LS2 3AA, United Kingdom; b Bradford Institute for Health Research, Bradford Teaching Hospitals NHS Trust, Bradford BD9 6RJ, United Kingdom; c Intermountain Healthcare Department of Population Health Sciences, Spencer Fox Eccles School of Medicine, University of Utah, Salt Lake City, UT 84112, USA; d Nuffield Department of Primary Care Health Sciences, University of Oxford, Oxford OX2 6GG, United Kingdom

**Keywords:** Antihypertensive, Blood pressure, Frailty, Hypertension, Multimorbidity, Aged pharmacotherapy

## Abstract

The authors conducted a review of pharmacologic therapy in older adults with hypertension. They reviewed the evidence supporting their use in older adults, understanding the physiologic changes and potential adverse drug effects associated with aging and antihypertensive medication use, exploring guideline recommendations for antihypertensive use in older adults, and evaluating the associated risks and benefits of specific classes of antihypertensive medications.

## Key points

• Absolute cardiovascular benefits of lowering blood pressure with antihypertensives increase with age.
• Across the population, physiological changes with age affect the pharmacokinetic and pharmacodynamic properties of antihypertensive medications. As a result, while the risk of experiencing harm from antihypertensive medications is low, it also increases with age.
• Prediction models are available to identify older adults who are at high risk of adverse events to inform personalized treatment.
• Most clinical guidelines recommend the “start low, go slow” approach for initiating antihypertensive medication among older adults with consideration of frailty and multimorbidity guiding choice of the antihypertensive agent.

## Introduction

In the United States, 3 out of 4 people will develop hypertension during their lifetime.^1^ Hypertension is the leading modifiable risk factor for cardiovascular disease which accounts for approximately 30% of all deaths worldwide, and older adults represent those at the highest risk.^2^, ^3^, ^4^ Nonpharmacological interventions are effective for lowering blood pressure (BP) yet difficult to achieve and maintain for most. Therefore, pharmacological therapy is usually needed to achieve BP control.

This article provides a review of the guidelines and the underlying evidence supporting recommendations for antihypertensive pharmacotherapy in older adults with hypertension. We review the most commonly used antihypertensive medication classes, relevant considerations for their use in older adults including the place for combination therapy, important adverse drug events (ADEs) to monitor and manage, and how to approach shared decision-making with older adults.

## Evidence supporting antihypertensive pharmacotherapy in older adults

**Fig. 1:**
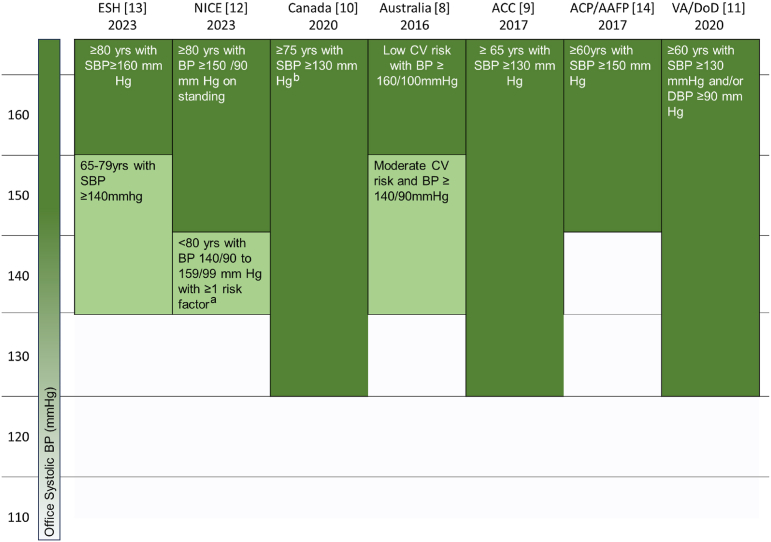
Age-based variations in guideline recommendations for initiation of antihypertensive pharmacotherapy based on systolic blood pressure measurements among non-frail, non-institutionalized persons. ^a^Additional risk factors include target organ damage, established atherosclerotic cardiovascular disease (ASCVD), renal disease, diabetes, estimated 10 year cardiovascular disease risk ≥10%. ^b^High-risk conditions include age ≥75 years, clinical or subclinical ASCVD, chronic kidney disease, or 10 year Framingham risk score ≥15%. ACC, American College of Cardiology; ACP, American College of Physicians; AAFP, the American Academy of Family Physicians; ESH, European Society of Hypertension; NICE, the National Institute for Health and Care Excellence; VA/DoD, Veterans Affairs/Department of Defense.

Increasing evidence supports the cardiovascular, renal, and neurovascular benefits of treating hypertension in older adults, emphasizing a thoughtful evaluation of risks and benefits.^5^, ^6^, ^7^ Of note, all of the professional society guidelines since 2016 endorse the use of pharmacotherapy in older adult populations with hypertension.^8^, ^9^, ^10^, ^11^, ^12^, ^13^, ^14^ However, guidelines vary widely on their age-based recommendations for thresholds for initiation and intensification of antihypertensive medication in older adults (Fig. 1).^15^

The decision on which antihypertensive medication to prescribe to reduce cardiovascular risk in older adults is generally perceived as less critical than achieving the target BP level. It is thought that the cardiovascular benefits result from the reduction in BP and less from direct effects of the medications independent of the BP change. There have been several randomized controlled trials (RCTs) of BP lowering with antihypertensive medication in older adults to inform treatment decisions (Table 1).^5^, ^6^, ^7^^,^^16^^,^^17^

**Table 1:** Randomized, controlled trials of antihypertensive medications in older adults with hypertension

Trial	Date	Achieved BP		Age Criteria	*N*	Intervention Arm Antihypertensive Medication Regimen	SBP Target Intervention vs comparator^a^	Median Follow-Up (Years)	Intervention vs Comparator (Hazard Ratio [95% CI] or *N* [*P* value])		
		Intervention	Comparator						All-Cause Mortality	MACE	ADE
HYVET^5^	2008	144/78	159/84	≥80 y	3845	TZD ± ACEI	TZD ± ACEI vs placebo	1.8	0.79 (0.65–0.95)	0.66 (0.53–0.82)	358 vs 448 (0.001)
JATOS^17^	2008	136/75	146/78	65–85 y	4418	CCB	<140 vs 140–<160	2	54 vs 42 (0.22)	26 vs 28 (0.78)	NR
bVALISH^16^	2010	137/75	142/77	70–84 y	3260	ARB first line	<140 vs 140–<150	3.1	0.78 (0.46–1.33)	0.84 (0.53–1.36)	281 vs 275 (0.85)
SPRINT-Senior^6^	2016	123/62	135/67	≥75 y	2636	TZD or ACEI/ARB or CCB first line	<120 vs <140	3.1	0.67 (0.49–0.91)	0.66 (0.51–0.85)	0.99 (0.89–1.11)
STEP^7^	2021	127/76	136/79	60–80 y	8511	ARB or CCB first line	110–<130 vs 130–<150	3.3	1.11 (0.78–1.56)	0.72 (0.56–0.93)	Hypotension^c^1.31 (1.02–1.68)

*Abbreviations:* ACEI, angiotensin-converting enzyme inhibitor; ADE, adverse drug event; AKI, acute kidney injury; ARB, angiotensin-II receptor blocker; BP, blood pressure; CCB, calcium channel blocker; CV, cardiovascular; HYVET, Hypertension in the Very Elderly Trial; JATOS, Japanese trial to assess optimal systolic blood pressure in elderly hypertensive patients; MACE, major adverse cardiovascular event; NR, not reported; SBP, systolic blood pressure; SPRINT, Systolic Blood Pressure Intervention Trial; STEP, strategy of blood pressure intervention in the elderly hypertensive patients; TZD, thiazidelike or thiazidtype diuretic; VALISH, valsartan in elderly isolated systolic hypertension study.

a Unit of measurement for BP is in mm Hg.

b Inclusion criteria was for only adults with isolated systolic hypertension (SBP > 160 and DBP < 90).

c Other ADEs including renal dysfunction were not significantly different between groups.

The Hypertension in the Very Elderly Trial (HYVET) and Systolic Blood Pressure Intervention Trial (SPRINT)-Senior are the two most widely cited RCTs to evaluate the effect of BP lowering in older adults. HYVET randomized patients age ≥80 years, with a sustained seated systolic BP (SBP) of 160 mm Hg or more, to receive either indapamide (with additional perindopril as required) or placebo.^5^ Patients in the treatment arm had a 15 mm Hg lower reduction in SBP than the placebo group which resulted in a reduced rate of major adverse cardiovascular events (MACE), hazard ratio (HR) 0.66, 95% confidence interval (95% CI) 0.53 to 0.82, and a lower rate of serious ADEs (*P* < .001). SPRINT-Senior was a prespecified subgroup analysis of those age ≥75 years at baseline in SPRINT which randomized US adults age ≥50 years at high cardiovascular disease risk, without diabetes mellitus or stroke, to intensive (SBP target <120 mm Hg) versus standard (SBP <140 mm Hg) control.^6^ In the age ≥75 years subgroup, participants in the intensive arm, compared to those in the standard treatment arm had a lower risk of MACE, HR 0.66, 95% CI 0.51 to 0.85 with no difference in the rate of serious ADEs HR 0.99, 95% CI 0.89 to 1.11.

In both HYVET and SPRINT-Senior, the investigators have examined the impact of frailty through retrospective analyses.^18^^,^^19^ In both trials, the effect of the randomized intervention on MACE or ADEs was not different across frailty levels at baseline. Importantly, the subgroup living with more advanced frailty in both HYVET and SPRINT-Senior demonstrated a benefit of BP-lowering interventions on mortality and cardiovascular disease outcomes but did not have a higher risk of ADEs. However, there is some debate as to whether older adults living with severe frailty were represented in the trial population.^20^

In a meta-analysis incorporating individual patient-level data (IPD) from 358,707 participants across 51 RCTs, the benefits of BP lowering with antihypertensive medication were assessed in two distinct age cohorts: 54,016 participants aged 75 to 84 years and 4,788 participants aged ≥85 years.^21^ The results demonstrated that each 5 mm Hg reduction in SBP consistently lowered the risk of MACE across all age groups, maintaining significance up to the age of 85 years. However, the magnitude of relative risk reduction varied by age, with the most substantial benefit observed in the youngest cohort and progressively smaller effects, accompanied by wider CIs, in older age groups. Specifically, the risks for MACE were as follows: HR 0.82, 95% CI 0.76 to 0.88 in adults less than 55 years; HR 0.91, 95% CI 0.88 to 0.95 for 55 to 64 years and similarly for 65 to 74 years; HR 0.91, 95% CI 0.87 to 0.96 for 75 to 84 years; and HR 0.99, 95% CI 0.87 to 1.12 for ≥85 years. There was some evidence suggesting that the treatment effect on MACE varied by age (adjusted *P* value for interaction = 0.050). The diminished relative effects observed in older age groups could be attributed to several factors. These include potentially shorter durations of treatment, a decreased ability to reverse cardiovascular risk as age progresses, and the presence of competing risks for cardiovascular disease. Another consideration is the simple issue of sample size; often, the number of older adults included in these trials is lower, leading to uncertain estimates of treatment effects in this subgroup. Conversely, the absolute risk reductions for MACE demonstrated variation across age groups, showing larger benefits in older populations (adjusted *P* value for interaction = 0.024). This pattern is likely explained by the higher baseline cardiovascular risk that accompanies advancing age, making even modest reductions in risk factors more impactful in terms of absolute risk reduction.

Applying the best available RCT evidence of the effect of lowering BP with antihypertensive medication to older adults is challenging for 3 predominant reasons:

• First, RCT populations are not fully representative of the target population. Trial designs that explicitly or implicitly exclude older adults with concurrent health issues result in highly selective trial populations. This selectivity is particularly evident in the exclusion of older adults with multiple health conditions, extensive medication use, frailty, and those residing in nursing homes.^22^
• Second, the outcomes measured in RCTs prioritize cardiovascular disease endpoints. There has only been limited enquiry, aside from the SPRINT trial, about the tolerability and degree to which treatment affects ADEs, daily function, and quality of life.
• Third, BP measurement and titration of antihypertensive therapy in a trial setting is not necessarily replicable in routine clinical care. Availability of routine follow-up, access to clinicians and medical staff, and medical resources for hypertension management are not readily available in a real-world context within the current health care landscape.

## Antihypertensive pharmacotherapy and adverse drug events in older adults

A systematic review of 58 RCTs found evidence that antihypertensive medication is associated with acute kidney injury (AKI) with a relative risk (RR) 1.18, 95% CI 1.01 to 1.39; hyperkalemia RR 1.89, 95% CI 1.56 to 2.30; hypotension RR 1.97, 95% CI 1.67 to 2.32; and syncope RR 1.28, 95% CI 1.03 to 1.59, but no evidence of an association with falls RR 1.05, 95% CI 0.89 to 1.24 or fracture RR 0.93, 95% CI 0.58 to 1.48.^23^ The lack of individual patient data in this review precluded an analysis of whether treatment effect varied by age. However, it is known that older adults are more susceptible to medication-related ADEs, in part due to altered physiology associated with aging.^24^ Older adults undergo many physiological changes which affect drug absorption, distribution, metabolism, and excretion (ADME).^25^ Table 2 provides a review of pharmacokinetic and pharmacodynamic ADME medication changes in older adults.

**Table 2:** Physiologic changes in pharmacokinetic and pharmacodynamic properties of antihypertensive medications associated with aging

Pharmacokinetic/Pharmacodynamic Property	Definition	Aging-related Changes
Absorption	Absorption of medication into systemic circulation from site of delivery (bioavailability)	1. Decreased gastric acid production, gastric motility, and small bowel surface area resulting in lower medication plasma concentrations. 2. Decreased skin hydration and lipophilicity possibly decreasing transdermal medication absorption (ie, clonidine patch)
Distribution	Distribution of medication into the blood stream and tissues	1. Increased body fat and decreased total body water resulting in increased plasma levels of water-soluble drugs and decreased plasma levels of lipophilic medications. 2. Changes in blood proteins which bind to free medication in the blood stream possibly altering free (ie, unbound) plasma concentrations, thereby, increasing or decreasing their effects and potential for toxicity
Metabolism	Breakdown of medication into water-soluble metabolites for elimination	1. Decreased liver mass, liver and splanchnic blood flow, and liver and intestinal enzyme activity resulting in decreased enzymatic transformation of medication before they reach systemic circulation (first-pass metabolism) 2. Decreased liver volume by up to 30% in older adults also reduces phase I metabolism of medications that interact via the cytochrome P450 enzymes
Excretion	Removal of medication or metabolites from the body (usually via the liver or kidney)	1. Decreased blood flow to the liver and the kidneys, and consequently, drugs which largely depend on blood flow for excretion (ie, drugs with a high “extraction ratio” of >0.7) may experience prolonged elimination times and greater risk for toxicity 2. Decreased kidney size, increased tubular fibrosis and atrophy, and reduced glomerular filtration rate due to hypertension or diabetes may increase plasma concentrations of renally excreted medications

In an observational study including 3.8 million patients in England aged ≥40 years with hypertension followed up over 10 years,^26^ new antihypertensive medication use was associated with an increased risk of hospitalization or death from falls with a HR 1.23, 95% CI 1.21 to 1.26; hypotension HR 1.32, 95% CI 1.29 to 1.35; syncope HR 1.20, 95% CI 1.17 to 1.22; AKI HR 1.44, 95% CI 1.41 to 1.47; electrolyte abnormalities HR 1.45, 95% CI 1.43 to 1.48; and a primary care visit with gout HR 1.35, 95% CI 1.32 to 1.37. Risks of ADEs rose with increasing age and frailty. The robustness of this observational analysis was tested by comparing the results to published estimates from the meta-analysis of RCTs cited earlier^21^ and found that the estimates of treatment effect fell within the 95% CIs of estimates from the meta-analysis of RCTs for all outcomes except hypotension and AKI.

## Guideline-directed antihypertensive pharmacotherapy in older adults

There are 11 classes of antihypertensive medications approved for BP lowering by the US Food and Drug Administration including alpha-blockers, alpha-receptor agonists, beta-blockers, peripheral adrenergic inhibitors, angiotensin-converting enzyme inhibitor (ACEIs), angiotensin-II receptor blockers (ARBs), direct renin inhibitors, aldosterone receptor antagonists, calcium channel blockers (CCBs), diuretics, and vasodilators. The potential role of novel antihypertensives currently in development (Table 3), in the treatment of hypertension in older adults, is currently unclear.^27^

**Table 3:** Novel antihypertensive medications currently in development

Medication	Mechanism of Action
Aldosterone synthetase inhibitors, for example, lorundrostat, baxdrostat	Blocks the synthesis of aldosterone and thereby prevents aldosterone-mediated sodium and water retention; increases in blood volume and elevated blood pressure.
Angiotensin receptor neprilysin inhibitor, for example, sacubitril/allisartan	Sacubitril blocks neprilysin, the enzyme responsible for breaking down natriuretic peptides. Prolonging activity of natriuretic peptides promotes vasodilation, natriuresis, and diuresis, thereby reducing blood pressure. Allisartan blocks angiotensin-2 receptors to counteract the accumulation of circulating angiotensin 2 caused by neprilysin inhibition.
Attenuators of hepatic angiotensinogen, for example, zilebesiran	Binds hepatic asialoglycoprotein receptor, preventing the formation of hepatic angiotensinogen and thereby blocking activation of the renin–angiotensin–aldosterone system.
Aminopeptidase A inhibitors, for example, firibastat	Inhibits conversion of angiotensin II to angiotensin III in the brain, thereby increasing diuresis and decreasing vasopressin levels, blood volume, sympathetic tone, and vascular resistance.
Atrial natriuretic peptide (ANP) analogs, for example, NCT03781739	Mimics endogenous ANP and inhibits renin and aldosterone, preventing angiotensin II-induced vasoconstriction.
Dual endothelin antagonists, for example, aprocitentan	Blocks endothelin-1 from binding to endothelin A and B receptors on vascular smooth muscle cells, blocking endothelin-1-mediated vasoconstriction, aldosterone synthesis, and catecholamine release.
Glucagonlike peptide-1 (GLP-1) receptor agonists, for example, tirzepatide	Unknown, but may be related to agonism of GLP-1 effects on natriuresis, direct vasodilation, sympathetic activation, or reductions in extracellular volume.

There are only 4 classes recommended as a first-line therapy to lower BP in older adults given their efficacy at preventing cardiovascular disease in RCTs: ACEIs, ARBs, CCBs, and thiazide-like or thiazide-type diuretics (TZDs; Table 4). Beta-1 selective adrenoreceptor antagonists also effectively prevent cardiovascular disease events but prevent stroke to a lesser degree than other agents and so are second-line agents unless a compelling indication is present. The remaining classes may be added to individualize regimens according to an individual’s comorbidities and compelling indications.

**Table 4:** Preferred antihypertensive medication classes to treat high blood pressure

		ARB/ACEI		CCB^b^	TZD	β-1 Selective Beta-blockers
Mechanism of action		Prevents angiotensin-II-mediated vasoconstriction, sodium retention, and water retention.		Vasodilatation by blocking Ca channels in vascular smooth muscle cells, limited chronotropic and inotropic effect.	Induce natriuresis and diuresis which reduces circulating blood volume.	Block sympathetic adrenergic transmission, negatively ionotropic and chronotropic.
Role in care		First line		First line	First line	Second-line (lower efficacy for preventing CV events)
Compelling indications		Stroke, heart failure, diabetes mellitus, CKD, stable CHD, postmyocardial infarction, aortic disease		Stable angina	Heart failure	Stable CHD/angina, postmyocardial infarction, HFrEF, atrial fibrillation
Contraindications		Severe bilateral renal artery stenosis History of angioedema with ACEIs.^c^		Severe aortic stenosis	Severe hepatic impairment, hypokalemia, COPD (relative)	Asthma, COPD with significant reversibility, heart block
ADEs in older adults	>1%	First-dose hypotension, cough,^a^ fatigue.		Pedal edema	Dose-dependent: hyponatremia, hypokalemia, hyperuricemia.	Fatigue, bradycardia, diminished exercise tolerance, impaired hypoglycemia awareness.
	<1%	Angioedema,^a^ acute kidney injury, hyperkalemia		Fatigue, hypotension	New-onset diabetes, hypercalcemia	New-onset diabetes, sleep disorders.
Risk of orthostatic hypotension		Low risk	Low risk	Low risk	Low risk^a^	Moderate risk
Harmful drug–drug interactions		Potassium supplements or potassium-sparing diuretics (hyperkalemia), renin inhibitors		CYP3A4 inhibitors, for example, Macrolide antibiotics. (accentuates effect)	Lithium (reduced lithium clearance).	Verapamil (severe hypotension and cardiac failure)
Renal impairment		Risk of hyperkalemia in established CKD			Switch to a loop diuretic if renal function <30 mL/min.	

*Abbreviations:* ACEI, angiotensin-converting enzyme inhibitor; ADE, adverse drug event; AKI, acute kidney injury; ARB, angiotensin receptor blocker; CCB, calcium channel blocker; CHD, coronary heart disease; CKD, chronic kidney disease; COPD, chronic obstructive pulmonary disease; CV, cardiovascular; HFrEF, heart failure with reduced ejection fraction; TZD, thiazide-like or thiazide-type diuretic.

a Low quality evidence.

b Dihydropyridine CCBs are more selective to the vasculature and are commonly used in all age groups.

c Patients with angioedema due to ACEI may trial an ARB 6 weeks after stopping ACEI.

The 2023 UK National Institute for Health and Care Excellence (NICE) guidelines distinctly endorse initial CCBs over TZDs in individuals aged 55 years and above based on cost-effectiveness data and lower variability in BP lowering, a factor linked to heightened cardiovascular risk.^28^^,^^29^ The 2020 Veterans Affairs/Department of Defense recommends TZDs as the first-line treatment in adults aged ≥65 years based on a meta-analysis demonstrating greatest protection against MACE reduction versus other first-line agents without evidence of increase ADEs.^11^^,^^30^

## Evidence for specific classes in older adults

**Fig. 2:**
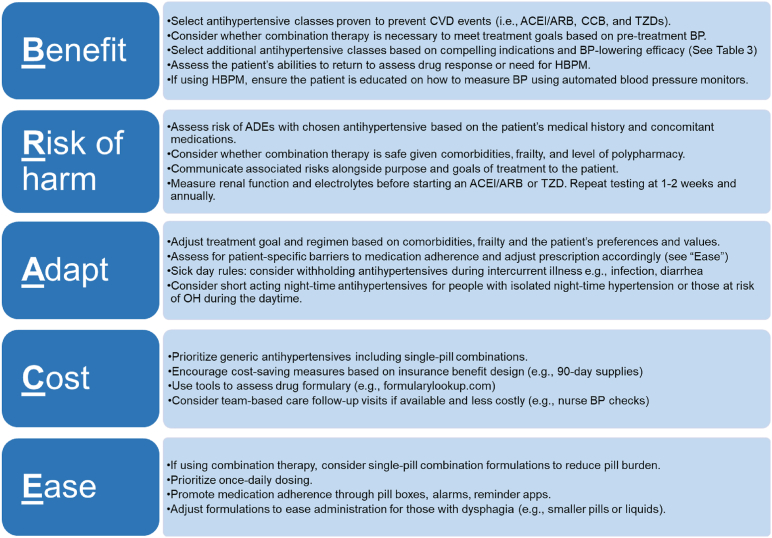
BRACE acronym to guide antihypertensive treatment selections for the older adult. ACEI, angiotensin-converting enzyme inhibitor; ADE, adverse drug event; ARB, angiotensin-II receptor blocker; BP, blood pressure; CCB, calcium channel blocker; CVD, cardiovascular disease; TZD, thiazidelike or thiazidetype diuretic.

A clinician’s selection of a particular agent may be influenced by factors such as other compelling indications, frailty, renal function, and the likelihood of experiencing ADEs.^31^ Each class of antihypertensive medication comes with nuanced and distinct advantages and disadvantages when used in older adults and these are considered in Table 4. We provide key factors to consider in initial treatment decisions via the BRACE acronym in Fig. 2, standing for Benefit, Risk of harm, Adapt, Cost, and Ease.

## Titration of therapy in older adults

Historically, clinical guidelines have recommended the “start low, go slow” approach for initiating antihypertensive medication among persons with advanced age, frailty, and multimorbidity. This approach involves starting one medication class at its lowest dose, increasing the dose slowly according to patient response, then adding another medication class at its lowest dose, increasing its dose slowly, and so on. This approach requires frequent patient follow-up to assess response and to titrate treatment, and the stepped care approach may increase therapeutic inertia, whereby one medication is initiated and remains unchanged thereafter despite evidence of ineffectiveness.^13^ Guidance is lacking on which practice is best for initiating and titrating therapy in older adults, including establishing specific thresholds to determine whether to use mono- or combination therapy.

Considerations on titrating therapy in older adults include

• The risk of first-dose hypotension and highest incidence of ADEs including falls presents in the first 1 to 2 weeks after starting treatment.^32^ A large case crossover study demonstrated increased risk of a serious fall injury after initiating an antihypertensive medication, odds ratio (OR) 1.36, 95% CI 1.19 to 1.55, adding a new class, and titration but these associations were not sustained beyond 15 days.^33^
• The requirement to measure standing BP and postural difference in BP to screen for orthostatic hypotension which may be exacerbated by certain classes of antihypertensives more than others.^34^ The 2017 American College of Cardiology/American Heart Association hypertension guideline also recommends screening for orthostatic hypotension in people during follow-up after initiation and in higher risk groups (eg, Parkinson’s disease and diabetes mellitus).^9^
• The need to check renal function and electrolytes at 1 to 2 weeks and annually thereafter in all first-line antihypertensive classes with the exception of CCBs, but also in the event of intercurrent illness which may increase the risk of electrolyte disturbance or renal failure and indicate the need for a short-term temporary pause of antihypertensive therapy until recovery.^12^

## Combination antihypertensive pharmacotherapy in older adults

Two medication classes each operating through different mechanisms initiated at low doses are more effective and tolerable than monotherapy initiated at standard or maximum doses.^35^^,^^36^ In younger patients, if the patient’s pretreatment SBP is ≥20 mm Hg (or DBP is ≥10 mm Hg) from their treatment goal, initiation of treatment using combination therapy may be indicated.^9^^,^^13^ There is uncertainty regarding the relative balance of effectiveness and safety of initiating monotherapy or combination therapy among older adults. Titration of therapy in the context of ADEs may be more difficult if these medications are started in combination rather than individually.

## Tailoring antihypertensive pharmacotherapy in older adults

**Fig. 3:**
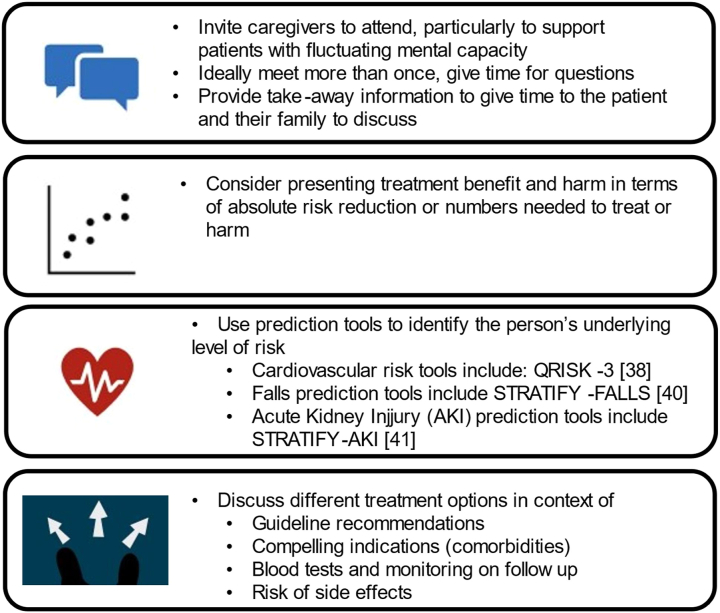
Step-by-step guide to shared decision-making in hypertension management in older adults.

Hypertension guidelines recommend shared decision-making which is an exercise in empowering a patient to be an agent in their medical care. The 2023 NICE hypertension guidelines offer a decision aid which may help older adults and their caregivers decide whether treatment is feasible for their situation.^12^ We recommend considerations relevant to the engagement of older adults in shared decision-making in relation to hypertension treatment (Fig. 3).

Presenting risk in terms of absolute risk differences has been shown to be better understood by patients and clinicians.^37^ Numbers needed to treat (NNTs) to prevent cardiovascular disease and numbers needed to harm (NNHs) represent an alternative means of presenting benefit and harm to communicate with patients to support shared decision-making about BP treatment. In Table 5, the NNT over 5 years has been calculated for different cardiovascular outcomes using data from an IPD meta-analysis of RCTs,^21^ alongside NNH over 5 years using data from a large routine data study.^26^ This could be used in discussions with patients to support shared decision-making about BP treatment. It is evident from this comparison that across the whole population, the likelihood of benefit from BP-lowering treatment was high, and the likelihood for experiencing harm was very low. However, the risk of benefit and harm becomes more balanced in older age groups. For example, for adults aged 80 to 89 years, prescription of a new BP-lowering treatment may be just as likely to cause a serious fall, as it would prevent a stroke or heart attack: the NNH for a serious fall over 5 years is estimated at 33,^26^ the NNT to prevent a major cardiovascular event over 5 years estimated at 23.^21^

**Table 5:** Numbers needed to treat and numbers needed to harm

		Numbers Needed to Treat (NNT) at 5 Years^19^					
Event		MACE	Stroke	Ischemic Heart Disease	Heart Failure	Cardiovascular Death	All-cause Mortality
Age Categories	65–74 y	38	120	55	100	301	100
	75–84 y	25	86	40	75	55	75
	85+ y	23	40	43	151	86	25

		Numbers Needed to Harm (NNH) at 5 Years^24^					
Event		Falls	Hypotension	Syncope	Acute kidney Injury	Electrolyte Abnormality	Gout
Age categories	60–69 y	400	222	250	100	118	125
	70–79 y	118	111	154	47	61	80
	80–89 y	33	56	167	27	27	105
	90+ y	20	51	69	16	19	95

NNTs were calculated over 5 years, as 1/absolute risk reduction associated with the mean blood pressure reduction using event rates in each category associated with treatment compared to control in data representing approximately 3 years follow-up, multiplied by 1.66 to approximate events over 5 years. NNHs were calculated over 5 years, as 1/absolute risk difference (additional events) using event rates over 5 years associated with a new antihypertensive prescription. Color coding is illustrative and does not represent agreed thresholds: for NNT: red >200; amber 100 to 199; green <100; for NNH: red <100; amber 100 to 199; green >200. MACE, major adverse cardiovascular event.

Absolute risk prediction is best understood with knowledge of a person’s baseline risk of the outcome. This can be estimated using prediction models. To identify patients who may benefit most from BP lowering, guidelines recommend estimating an individual’s cardiovascular risk using validated tools (eg, QRISK3,^38^ PREVENT [American Heart Association Predicting Risk of CVD Events]^39^). Equivalent tools now exist also for identifying a person’s risk of developing ADEs related to antihypertensive therapy, specifically their risk of falls (STRATIFY-Falls)^40^ and risk of developing AKI (STRATIFY-AKI).^41^^,^^42^

## Summary

In contrast to historical practices, there are now strong evidence and guideline recommendations endorsing the use of antihypertensive medications in older adults with hypertension. Employing shared decision-making between the clinician and patient is crucial. This process should involve careful consideration of physiological changes with age, the risk of ADEs, and an individualized assessment of the benefits and harms when deciding to initiate or select specific antihypertensive medication therapy for older adults.

## Clinics care points

• Evidence supports the cardiovascular and neurovascular benefits of treating hypertension in older adults.
• Across the whole population, the likelihood of benefit from BP-lowering treatment is high, and the likelihood for experiencing harm is very low. However, the risk of benefit and harm becomes more balanced in older age groups.
• Choice of a particular agent should consider compelling indications, concomitant medications, renal function, and the likelihood of experiencing adverse drug events.
• Employing shared decision-making between the clinician and patient is crucial.
• Presenting risk in terms of absolute risk differences is more easily understood by patients and clinicians and best undertaken with knowledge of a person’s baseline risk of the outcome.
• Prediction models are available to identify older adults who are at high risk of cardiovascular disease and equivalent tools now also exist for identifying a person’s risk of developing adverse drug events related to antihypertensive therapy.
• We recommend the “start low, go slow” approach for initiating antihypertensive medication among older adults.
